# Geographic distribution of the incidence of colorectal cancer in Iran: a population-based study

**DOI:** 10.4178/epih.e2017020

**Published:** 2017-05-17

**Authors:** Fatemeh Khosravi Shadmani, Erfan Ayubi, Salman Khazaei, Mohadeseh Sani, Shiva Mansouri Hanis, Somayeh Khazaei, Mokhtar Soheylizad, Kamyar Mansori

**Affiliations:** 1 Modeling in Health Research Center, Institute for Futures Studies in Health, Kerman University of Medical Sciences, Kerman, Iran; 2 Department of Epidemiology, School of Public Health, Shahid Beheshti University of Medical Sciences, Tehran, Iran; 3 Department of Epidemiology and Biostatistics, School of Public Health, Tehran University of Medical Sciences, Tehran, Iran; 4 Department of Epidemiology, School of Public Health, Hamadan University of Medical Sciences, Hamadan, Iran; 5 School of Medicine, Zabol University of Medical Sciences, Zabol, Iran; 6 School of Public Health, Dezful University of Medical Sciences, Dezful, Iran; 7 Department of Para Medicine, Hamadan University of Medical Sciences, Hamadan, Iran; 8 Social Determinants in Health Promotion Research Center, Hormozgan University of Medical Sciences, Bandar Abbas, Iran; 9 Social Development and Health Promotion Research Center, Gonabad University of Medical Sciences, Gonabad, Iran; 10 Department of Epidemiology, School of Public Health, Iran University of Medical Sciences, Tehran, Iran

**Keywords:** Colorectal neoplasms, Sex, Incidence, Epidemiologic studies, Iran

## Abstract

**OBJECTIVES:**

Colorectal cancer (CRC) is the third most common cancer and the fourth most common cause of cancer death in the world. The aim of this study was to investigate the provincial distribution of the incidence of CRC across Iran.

**METHODS:**

This epidemiologic study used data from the National Cancer Registry of Iran and the Center for Disease Control and Prevention of the Ministry of Health and Medical Education of Iran. The average annual age-standardized rate (ASR) for the incidence of CRC was calculated for each province.

**RESULTS:**

We found that adenocarcinoma (not otherwise specified) was the most common histological subtype of CRC in males and females, accounting for 81.91 and 81.95% of CRC cases, respectively. Signet ring cell carcinoma was the least prevalent subtype of CRC in males and females and accounted for 1.5 and 0.94% of CRC cases, respectively. In patients aged 45 years or older, there was a steady upward trend in the incidence of CRC, and the highest ASR of CRC incidence among both males and females was in the age group of 80-84 years, with an ASR of 144.69 per 100,000 person-years for males and 119.18 per 100,000 person-years for females. The highest incidence rates of CRC in Iran were found in the central, northern, and western provinces. Provinces in the southeast of Iran had the lowest incidence rates of CRC.

**CONCLUSIONS:**

Wide geographical variation was found in the incidence of CRC across the 31 provinces of Iran. These variations must be considered for prevention and control programs for CRC, as well as for resource allocation purposes.

## INTRODUCTION

Colorectal cancer (CRC) is the third most common cancer in the world after lung cancer and breast cancer. It is also fourth most common cause of cancer death globally [1]. In males, CRC is the third most commonly diagnosed cancer worldwide, and in females, it is the second most commonly diagnosed cancer. In 2012, more than 9% of all new cases of cancer were attributed to CRC, accounting for roughly 1.4 million cases [2]. Of these cases, 746,000 were in males and 614,000 were in females [3].

The incidence of CRC is not distributed uniformly around the world, with the occurrence of CRC differing at least 25-fold from country to country [4]. A high incidence of CRC has been observed in high-income countries, such as the US, New Zealand, and Canada, and developing countries also continue to experience increasing incidence rates [5]. The highest increase in rates has been observed in Asian countries [6]. In Iran, where CRC is the fourth most common type of cancer (the third most common in females and the fifth most common in males), the incidence of the disease has experienced the same increase as those of other Asian countries, with CRC accounting for 8.4% of all cancers in the country [7]. One study found that the burden of CRC in Iran, based on the disability-adjusted life years index, was 52,53 years [8].

Inflammatory bowel disease, family history of CRC, obesity, diet, smoking, physical inactivity [9], and diabetes [10] are well-known risk factors for CRC. Furthermore, the incidence varies across different geographic areas due to environmental, social, and behavioral factors, with environmental risk factors having been found to play the most important role in the incidence and development of CRC [9]. Consequently, individuals within the same or adjacent areas that tend to share the same ethnic and cultural characteristics may be exposed to the same risk factors.

There are many ethnic groups in Iran that are subject to a variety of different risk factors. This study aimed to describe the geographic differences in the incidence of CRC across the country. Using these findings, we can provide policy makers and planners with advice on resource allocation, screening programs, and treatment strategies for various provinces across Iran.

## MATERIAL AND METHODS

This epidemiologic study used data from 2008 that were collected at the provincial level from the National Cancer Registry of Iran and the Center for Disease Control and Prevention of the Ministry of Health and Medical Education of Iran [11]. Patient data were originally collected by the Iranian Cancer Registry, which collects only pathology-based records from pathological laboratories across the country. Hospital-based and death certificate-based data were not included.

Data collected through the Iranian Cancer Registry were transmitted to the Iranian Ministry of Health every 3-month after repeated records had been removed. The registered data were then classified into 3 parts: part I included patient characteristics, such as age, sex, race, and place of residence; part II included the clinical history of the patient; and part III included preclinical findings. Data on the primary location of the tumor, diagnosis, morphology, cancer histology and behavior, and diagnostic method were registered accordingly.

In part I, the name of the physician who performed the biopsy, the name of the hospital where the biopsy occurred, the location from which the biopsy was taken, the clinical diagnosis, and the date the biopsy was sent to the histology laboratory were included, in addition to general patient characteristics, such as demographic information such as race and place of residence. In part II, the most important findings from patients’ clinical records were included. In part III, preclinical findings were included. Physicians filled out the clinical data form while the official personnel recorded personal and demographic information in accordance with the International Classification of Diseases for Oncology (ICD-OC; topography with ICD-OM: morphology). Data were collected manually and analyzed by age and sex.

### Statistical analysis

For each province, the average annual age-standardized rate (ASR) per 100,000 person-years was calculated for the incidence of CRC using the World Health Organization world standard population [11]. The data were analyzed using MS Excel 2010 (Microsoft Corp., Redmond, WA, USA). ArcGIS 10.4.1 (ESRI, Redlands, CA, USA) was used for mapping the incidence of CRC per province.

## RESULTS

**Figure 1.**
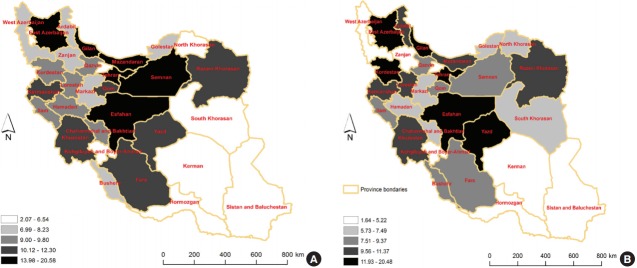
The geographic distribution of the age-standardized rate of colorectal cancer incidence in Iran, by sex (A: male, B: female).

Overall, 6,185 cases of CRC were registered in 2008. An assessment of the geographic distribution of these cases showed that the highest CRC incidence rates in Iran were in central, northern, and western provinces, respectively. In the central provinces of Tehran and Semnan, the ASRs of CRC incidence were 20.58 and 18.91 per 100,000 person-years, respectively. The southeast provinces of Iran had the lowest incidence rates of CRC (Figure 1).

The results of this study showed that adenocarcinoma (not otherwise specified) was the most common subtype of CRC in both sexes, accounting for 81.91% of CRC cases in males and 81.95% of CRC cases in females. Signet ring cell carcinoma was the least common subtype of CRC in males and females, accounting for 1.50 and 0.94% of CRC cases, respectively (Table 1).

**Figure 2.**
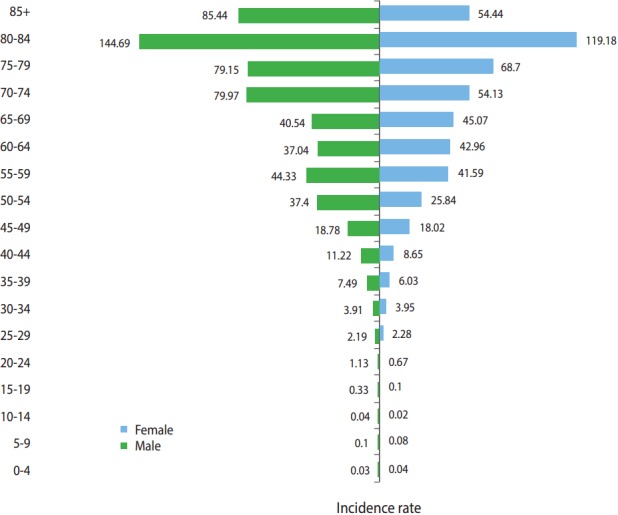
Age-standardized rates of colorectal cancer incidence in 2008, by age group and sex.

Figure 2 shows the age-specific and sex-specific incidence of CRC. The asymmetric shape of the pyramid indicates that the incidence of CRC was higher in those who were aged 45 years or older. The highest ASRs of CRC incidence in both sexes were in the age group of 80-84 years, with 144.69 per 100,000 personyears and 119.18 per 100,000 person-years in males and females, respectively.

The frequency, ASR, crude rate, and rank of CRC among other cancers for each province are shown in Table 2 and are stratified by sex. For Iranian males, Tehran had the highest ASR of CRC incidence, with 20.58 per 100,000 person-years, and Sistan and Baluchestan had the lowest ASR of CRC incidence, with 2.07 per 100,000 person-years. In addition, Gilan and Sistan and Baluchestan had the highest ASRs of CRC incidence among Iranian females, with 20.48 and 1.64 per 100,000 person-years, respectively. CRC was also among the 10 most common cancers among males and females in all provinces of Iran, and was the fourth most common cancer in males and the third most common cancer in females (Table 2).

## DISCUSSION

The highest incidence rates of CRC in Iran were found in the central, northern, and western provinces. The southwest provinces of Iran had the lowest incidence rates of CRC in the country. The incidence of CRC was higher in males than in females and increased with age. However, within the age groups of 60-64 years and 65-69 years, the incidence rates for females were higher than the incidence rates for males, and in males aged 70 years or older, the incidence of CRC was the highest. Additionally, adenocarcinoma (not otherwise specified) was the most common subtype of CRC in both sexes.

Variations in CRC incidence rates were observed after adjusting for age across different provinces in the country. The highest incidence of CRC occurred in provinces located around central and northern Iran. Some studies found that incidence rates of CRC were not uniformly distributed across different geographic areas [12-14]. Other reports also found that the ASR of CRC incidence in southern Iran was lower than the ASR in northern Iran [1,15,16], which is consistent with our results. In different studies, several aspects of the variation in incidence rates were investigated.

The role of socioeconomic status (SES) must be considered. The association between SES and the incidence of CRC differs across the globe [17]. Some studies reported that people living in low income areas had lower incidence rates of CRC than those living in higher income areas and that SES was significantly associated with CRC incidence [13,18]. On the contrary, individuals with lower SES were found to have a higher incidence of CRC in North America [19] and Canada [20].

There are several mechanisms through which the incidence of CRC might differ across geographic areas. Firstly, changes in lifestyle and differences in exposure to risk factors are the consequence of SES over time. Diet, obesity, and physical inactivity, as lifestyle characteristics, might play crucial roles in the etiology of CRC [21]. According to a previous estimation, almost 45% of all cases of CRC in high-risk populations could be attributed to these risk factors [22].

Secondly, high SES has been found to be related to high screening participation, which may lead to an increase in the incidence of CRC due to early detection of the disease [23]. However, high levels of screening may result in a decrease in the incidence of CRC if they lead to a rise in the removal of tumors at a precancerous stage [24]. Furthermore, disparities in access to health services and therapeutic interventions may be associated with SES and, subsequently, geographic variation in the disease [25].

Lastly, an increase in the incidence of CRC has been reported in industrial regions that have high concentrations of carcinogens in the air, water, and soil [26].

Our findings are consistent with the current literature. Iran’s population consists of various ethnic groups and cultural systems that vary by region. Regions with higher standards of living and better SES were found to have higher incidence rates. These areas were also more industrial than others. Furthermore, the prevalence of risk factors related to CRC was found to be higher in these regions.

Previous studies have emphasized the importance of geographical factors on CRC. Some studies have found an association between vitamin D deficiency and the incidence of CRC [22,27,28], with vitamin D levels varying by region. Subsequently, regions with low sunlight intensity have been found to have a high incidence of CRC. Our study corroborates these findings, as the lowest ASRs for CRC incidence were found in the southern provinces of the country that are closer to the equator.

We found that the ASR of CRC incidence in males was higher than in females, with a male-to-female ratio of 1.14:1. In China, this ratio was found to be 1.42:1 [4], and in Malaysia, this ratio was found to be 1.33:1 [16]. The male-to-female incidence ratio in the US was found to be 1.1 for patients aged 49 years or younger, 1.4 for patients aged 50-79 years, and 1.2 for patients aged 80 or older [7]. Studies have shown that the incidence of CRC in females may be reduced by hormone replacement therapy through the effect of female sex hormones on cholesterol metabolism, which may subsequently affect bile acid production. This pathway has been associated with the development of CRC [10].

We found that the incidence of CRC increased with age, particularly after the age of 50. It has been reported that CRC is not common among individuals aged 40 years or younger [29] and that incidence increases after the age of 40, and more sharply, after the age of 50 [2]. This is consistent with our findings.

The relationship between age and CRC is yet to be understood [30]. This may be due primarily to the time-dependent accumulation of risk factors that leads to genetic and epigenetic mutations [31]. The disruption of DNA repair mechanisms and cell growth regulation systems, increased inflammation, and decreased immune system function occur with aging and may lead to genetic mutations. Our results, however, suggest that the optimal age for screening is 50 years old.

In Iran, the most common subtype of CRC was found to be adenocarcinoma (not otherwise specified). Studies conducted in China [32] and Jamaica [33] found similar results.

One important strength of our study was the use of routine data from the Iranian Cancer Registry, which covered 86.7% of the population. There were, however, some limitations to our research. We had little access to data on cancer staging at the time of diagnosis, which limited our ability to conduct a more detailed investigation. However, this limitation did not have any effect on the results of our data on the provincial level.

In conclusion, wide geographical variation was found in the incidence of CRC across the 31 provinces of Iran. These variations must be considered for prevention and control programs for CRC, as well as for resource allocation purposes.

## Figures and Tables

**Table 1.** Morphological subtypes of colorectal cancer in 2008, by sex

	Adenocarcinoma NOS	Neoplasm, malignant	Mucinous adenocarcinoma	Mucin-producing adenocarcinoma	Signet ring cell carcinoma
	
Male	1,794 (81.91)	84 (3.83)	97 (4.42)	60 (2.73)	33 (1.50)
Female	1,381 (81.95)	75 (4.45)	74 (4.39)	49 (2.94)	16 (0.94)

Vales are presented as number (%).

NOS, not otherwise specified.

**Table 2.** Colorectal cancer statistics according to sex and province

Province	Male				Rank	Female				Rank	Population of province
	Fre	CR	ASR	%		Fre	CR	ASR	%		
East Azerbaijan	216	11.70	14.97	8.46	4	163	9.30	13.32	8.18	4	3,724,620
Fars	178	8.11	10.47	7.40	6	124	5.95	8.61	6.28	3	4,596,658
Razavi Khorasan	265	9.01	11.82	7.57	5	216	7.74	11.37	7.75	4	5,994,402
Esfahan	248	10.51	13.98	9.38	3	184	8.22	11.93	8.39	3	5,779,312
Gilan	153	12.54	16.35	10.92	4	163	14.08	20.48	15.75	2	2,480,874
Kermanshah	86	8.96	11.55	8.41	4	77	8.46	11.26	9.41	3	1,945,227
West Azerbaijan	82	5.61	7.37	6.78	5	47	3.39	5.22	5.38	6	3,080,576
Semnan	43	14.25	18.91	8.96	3	17	5.94	9.37	4.51	6	631,218
Kordestan	60	8.11	9.80	6.05	7	57	8.12	12.67	7.95	5	1,493,645
Yazd	49	9.66	12.30	7.57	5	51	10.60	14.96	8.84	4	1,074,428
Chaharmahal & Bakhtiari	33	7.52	9.00	8.38	5	17	4.08	6.09	6.16	6	895,263
Hamadan	63	7.24	9.66	5.90	6	35	4.24	5.84	5.23	5	1,758,268
Qazvin	40	6.89	9.36	7.50	4	35	6.35	9.11	8.14	3	1,201,565
Zanjan	30	6.11	6.99	7.79	4	12	2.58	3.46	4.84	5	1,015,734
Ilam	18	6.45	9.20	8.49	5	17	6.42	9.14	11.56	4	557,599
Kohgiluyeh & Boyer-Ahmad	15	4.63	6.54	5.81	6	10	3.25	4.45	5.62	6	658,629
Mazandaran	163	10.96	14.46	9.16	4	111	7.86	11.07	7.46	4	3,073,943
Tehran	1,096	16.03	20.58	10.46	5	774	11.93	16.84	8.95	3	12,183,391
Lorestan	61	7.01	9.48	6.30	6	54	6.54	9.56	7.62	5	1,754,243
Khuzestan	192	8.82	11.65	7.19	7	145	7.02	10.02	6.03	3	4,531,720
Markazi	43	6.19	8.23	7.49	5	33	5.01	6.93	8.55	3	1,413,959
Golestan	49	5.93	7.97	7.90	5	41	5.23	7.49	8.06	3	1,777,014
Qom	42	7.85	10.12	10.24	3	32	6.31	8.86	9.47	3	1,151,672
Ardabil	41	6.53	8.08	5.99	6	38	6.37	9.81	8.54	4	1,248,488
Kerman	59	4.41	5.69	5.51	7	44	3.47	4.83	5.17	5	2,938,988
North Khorasan	13	3.13	3.75	4.35	7	18	4.54	6.28	7.23	4	867,727
Bushehr	24	5.21	7.33	8.39	4	24	5.50	7.51	8.76	3	1,032,949
Hormozgan	34	4.86	5.78	11.30	1	22	3.32	4.21	7.28	2	1,578,183
Sistan & Baluchestan	20	1.63	2.07	5.85	5	14	1.20	1.64	4.50	7	2,534,327
South Khorasan	7	2.44	3.06	3.61	10	11	4.05	5.73	5.91	6	662,534
Total	3,527	9.80	12.70	8.34	5	2,658	7.78	11.12	7.85	3	73,637,156

Fre, frequency; CR, crude rate; ASR, age-standardized rate.
